# *Cucurbita argyrosperma* Seed Extracts Attenuate Angiogenesis in a Corneal Chemical Burn Model

**DOI:** 10.3390/nu11051184

**Published:** 2019-05-27

**Authors:** María Fernanda Estrella-Mendoza, Francisco Jiménez-Gómez, Adolfo López-Ornelas, Rosa Martha Pérez-Gutiérrez, Javier Flores-Estrada

**Affiliations:** 1 Laboratorio de Investigacíon de Productos Naturales, Escuela de Ingeniería Química e Industrias Extractivas, Instituto Politécnico Nacional, Unidad Profesional Adolfo López Mateos, Av. Instituto Politécnico Nacional S/N, Ciudad de México 07708, México; fernandaestrella29@hotmail.com (M.F.E.-M.); rmpg@prodigy.net.mx (R.M.P.-G.); 2 División de Investigación, Hospital Juárez de México, Av. Instituto Politécnico Nacional 5160, Magdalena de las Salinas, Gustavo A. Madero, Ciudad de México 07760, México; microcirugiafco@hotmail.com (F.J.-G.); adolfolopezmd@gmail.com (A.L.-O.)

**Keywords:** *Cucurbita argyrosperma*, corneal chemical burn, angiogenesis, corneal neovascularization (CNV), vascular endothelial growth factor (VEGF), interleukin-1β (IL-1β), cyclooxigenase-2 (COX-2), nuclear factor-kappaB (NF-κB)

## Abstract

Severe corneal inflammation produces opacity or even perforation, scarring, and angiogenesis, resulting in blindness. In this study, we used the cornea to examine the effect of new anti-angiogenic chemopreventive agents. We researched the anti-angiogenic effect of two extracts, methanol (Met) and hexane (Hex), from the seed of *Cucurbita argyrosperma*, on inflamed corneas. The corneas of Wistar rats were alkali-injured and treated intragastrically for seven successive days. We evaluated: opacity score, corneal neovascularization (CNV) area, re-epithelialization percentage, and histological changes. Also, we assessed the inflammatory (cyclooxigenase-2, nuclear factor-kappaB, and interleukin-1β) and angiogenic (vascular endothelial growth factor A, VEGF-A; -receptor 1, VEGFR1; and -receptor 2, VEGFR2) markers. Levels of *Cox-2*, *Il-1β*, and *Vegf-a* mRNA were also determined. After treatment, we observed a reduction in corneal edema, with lower opacity scores and cell infiltration compared to untreated rats. Treatment also accelerated wound healing and decreased the CNV area. The staining of inflammatory and angiogenic factors was significantly decreased and related to a down-expression of *Cox-2*, *Il-1β*, and *Vegf*. These results suggest that intake of *C. argyrosperma* seed has the potential to attenuate the angiogenesis secondary to inflammation in corneal chemical damage.

## 1. Introduction

Angiogenesis, inflammation, and oxidative stress are important factors that predispose to and promote the progression of degenerative diseases, and corneal diseases are not an exception. Corneal neovascularization (CNV) caused by viral infections, autoimmune diseases, and chemical burns can progress to corneal ulceration and scaring, potentially leading to blindness. CNV is also associated with a high rate of corneal allograft rejection [1]. In this context, the nuclear factor-kappaB (NF-κB) signaling pathway in inflammatory, epithelial, and endothelial cells is a key step for the transcriptional overexpression of pro-inflammatory and proangiogenic factors, including interleukin-1β (IL-1β), cyclooxygenase-2 (COX-2), and vascular endothelial growth factor A (VEGF-A). The cyclooxigenase-2 enzyme increases the synthesis of prostaglandins to modulate cell proliferation, cell death, and tumor invasion in many types of cancer. Interleukin-1β, IL-6, and tumor necrosis factor alpha (TNF-α) regulate COX-2; therefore, they are overexpressed during inflammation [2,3,4].

In an alkali-burn corneal injury, VEGF-A is an important aspect of angiogenesis expressed in macrophages and epithelial cells. Vascular endothelial growth factor A binds to two main tyrosine kinase receptors, vascular endothelial growth factor receptor 1 (VEGFR1) and –receptor 2 (VEGFR2), on a vascular endothelial cell to promote migration, proliferation, and neovascularisation, along with monocyte/macrophage migration in the microenvironment injured [1,5,6]. Several therapeutic strategies to decrease CNV have been used, such as topical or subconjunctival corticosteroids and non-steroidal anti-inflammatory agents [7]. However, these have limited use and have a number of side effects [8,9]. Anti-VEGF therapy in chemically burned ocular tissues results in a substantial reduction of angiogenesis in both animal studies and clinical trials [10,11]. However, to establish its safety and efficacy, controlled and randomized trials to justify their continued use are required. Besides, systemic drug treatment is not recommended because of adverse effects. Therefore, it is important to search for new drugs for the systemic treatment of these disorders.

Current data in CNV models show that natural extracts from plants or bioactive compounds in plant extracts have angiogenic suppressing activity [12,13,14]. The genus *Cucurbita* (pumpkin) belongs to one of the 300 genera of the Cucurbitaceae family, and it is one of the most popular vegetables eaten in the world. Recently, pumpkin was recognized as a functional food, and *Cucurbita pepo*, *Cucurbita maxima*, *Cucurbita moschata*, *Cucurbita andreana*, and *Cucurbita ficifolia* are the most cultivated species [15]. Nutritionally, pumpkin seed has a high amount of polyunsaturated fatty acids as well as proteins, vitamins, several minerals, and other phytochemicals. The anti-diabetic, antioxidant, anti-carcinogenic, and anti-inflammatory properties of this seed are studied due to its high content of natural bioactive compounds, such as carotenoids, tocopherols, and sterols [16,17,18,19].

*Cucurbita argyrosperma* is an economically important species cultivated in Mesoamerica. Isozyme, morphological, and ecological analyses suggest that it was probably domesticated from the Mexican wild squash, *Cucurbita sororia* [20]. The seed is usually consumed as a snack or as an ingredient in traditional stews, although the scientific findings of its beneficial effects on human health have not been sufficiently evidenced, and the anti-neovascular effects of the secondary metabolites remain unknown. However, conceivably its phytochemical composition could be like the related species, showing anti-inflammatory effects as suggested. Besides, proangiogenic factors such as COX-2, IL-1β, and VEGF, including VEGFR1 and -R2, induced by the inflammatory agents have not been studied in these plants.

The aim of this study was to investigate the effect of the seed extracts from *C. argyrosperma* in the inflammatory and angiogenic process of attenuation. Herein, we show that hexanic and methanolic extracts from *C. argyrosperma* seed significantly attenuate the expression of proangiogenic factors during inflammation using a CNV model. Also, we observed by clinical manifestation that both extracts significantly diminish the CNV area. Significantly, corneal re-epithelialization was higher with hexane (Hex) extract treatment than methanol (Met) extract.

## 2. Materials and Methods

### 2.1. Extract Preparation

Pumpkins of *C. argyrosperma* were harvested in an agricultural field of Michoacán, México, and identified by a botanist in the herbarium of the National Polytechnic Institute (IPN). Voucher specimen number 4532 was deposited in the herbarium of the National School of Biological Sciences of IPN. One kilogram of seed was extracted with 3 L of hexane (50% *v*/*v*) and left to macerate for 8 days at room temperature. The crude extract was filtered for one hour in 8 μM-medium flow filter paper (Whatman^®^, Sigma-Aldrich, Inc., St. Louis, MO, USA), concentrated using a rotary vacuum evaporator and taken to dryness at 60 °C in a vacuum rotator until the complete removal of the solvent, obtaining a viscous residue (8.38 g/L). The same procedure was applied to the residue, using methanol for a sequential separation of the seed components. Each extract was stored in the dark at 4 °C until use.

### 2.2. Animal Model

Twenty-eight male Wistar rats weighing 200−250 g (12 weeks old) were used. Water and standard food were available ad libitum. The care and management of experimental animals were in accordance with the guidelines of the National Institutes of Health “Guide for the Care and Use of Laboratory Animals”, the standards described by the Association for Research in Vision and Ophthalmology (ARVO), and Official Mexican Standard NOM-062-ZOO-1999. The study was approved by the Ethics Committee of the Hospital Juárez de México (HJM 2493/14-B).

### 2.3. Experimental Design

Animals were intraperitoneally anesthetized with pentobarbital sodium (0.5 mg/kg), inhaled sevoflurane, and one drop of ophthalmic tetracaine, to perform the chemical cauterization of the cornea. Central corneas from the right eyes were burned by applying 3-mm-diameter filter paper saturated with 1M NaOH solution for 30 s, with immediate washing with 10 mL of saline solution. To avoid infection, a drop of ophthalmic ciprofloxacin was applied every 24 h until the end of the study [21]. Each extract was dissolved in water and Tween-80 (20%) (Veh). Rats were randomly divided into four groups (*n* = 7 each) [21,22]: non-chemically burned healthy corneas (non-CB) treated only with the vehicle; chemically burned (CB) corneas treated with the vehicle (CB-Veh); CB corneas treated with hexanic extract (CB-Hex); and CB corneas treated with methanolic extract (CB-Met). All groups received 400 mg/kg/7 days of Hex/Met extracts or the vehicle in a single dose (0.5 mL) by oral gavage, at the same time daily (10:00 h). Animals were euthanized with an overdose of pentobarbital.

### 2.4. Clinical Manifestation

Corneal opacity, epithelial defects, and the CNV area were evaluated eight days after CB. Corneal opacity was scored using a scaling system from 0 to 4: 0 = no opacity, completely clear cornea; 1 = slightly hazy, iris and lens visible; 2 = moderately opaque, iris and lens still visible; 3 = severely opaque, iris and lens hardly visible; and 4 = completely opaque, with no visibility of the iris and lens [23].

The measurement of the CNV area (mm^2^) was performed in vivo using a ruler under a microscope and photographed. The software program Image-Pro Plus version 6.0 software (by Media Cybernetics, Inc., Rockville, MD, USA) was used. Inferonasal quadrant was selected to calculate the neovascularized area, according to a previous report [24].

To evaluate corneal wound re-epithelialization, we used corneal fluorescein staining. Briefly, fluorescein sodium ophthalmic strip was instilled into the lower conjunctival sac and the cornea was examined using a slit lamp biomicroscope with cobalt blue light. Injured epithelial tissues retain the fluorescein staining, whereas the lack of stain indicates re-epithelialization. The re-epithelialization percentage was evaluated in the CB corneas, considering that a total area of approximately 7 mm^2^ was 100 percent (a 3-mm disc saturated with 1M NaOH).

### 2.5. Histological Evaluation

Enucleated eyes (*n* = 4 per group) were immediately fixed in neutral formalin. Cut tissue slides (3–5 mm) were made (anteroposterior) and included the optic nerve. Slides were dehydrated in graded alcohols and embedded in paraffin. Histological sections of 2 μm were processed and stained with hematoxylin–eosin (HE). We measured the corneal thickness and cell infiltration in the peripheral region (500 µm beyond the limbus) using light microscopy (axioscope 2 plus, Carl Zeiss, Göttingen, Germany). The percentage of the infiltration was calculated in a masked fashion based on the density in the corneal stroma of the CB-Veh group.

Other 2-μm-sections were dewaxed and rehydrated with antigen recovery solution (ImmunoDNA Retriever 20× with Citrate; BioSB, Santa Barbara, CA, USA). Slides were then loaded into a Shandon Sequenza chamber (Thermo Shandon, Cheshire, United Kingdom). We used the procedure described for the polymer-based immunodetection system (PolyVue^®^ mouse/rabbit 3,3′-Diaminobenzidine, DAB, detection system, Diagnostic BioSystems, Pleasanton, CA, USA). We applied 100 μL of IL-1β (Cat. No. sc-7884), NF-κB p65 (sc-8008), COX-2 (sc-1746), VEGF-A (sc-7269), and VEGFR1 (sc-31173). All antibodies were purchased from Santa Cruz Biotechnology, Inc. (Santa Cruz, CA, USA). The VEGFR2 antibody (MAB3571) was purchased from R&D Systems, Inc. (Minneapolis, MN, USA). All dilutions were at 1:200 and incubated overnight at 4 °C. Later, the enhancers Polyvue Plus and HRP were added and incubated with DAB plus/chromogen substrate and counterstained with hematoxylin. An Axio Imager.A2 microscope with an integrated camera, Axiocam ICc5, (Carl Zeiss Microscopy GmbH, Jena, Germany) was used for histological observation and image capture. Micrographs of the peripheral region of the cornea (three fields per side at 200× magnification) were taken to measure the mean staining intensity of these markers. Images were analyzed with the Image-Pro Plus software version 6.0 (Media Cybernetics, Inc., Rockville, MD, USA).

### 2.6. Quantitative Reverse-Transcription Polymerase Chain Reaction (qRT-PCR)

Total RNA was isolated from corneal tissue using TRIzol™ Reagent (Invitrogen, Boston, MA, USA) (*n* = 3 per group). One microgram of DNase I-treated RNA (Roche Applied Science, Mannheim, Germany) was reverse transcribed with SuperScript^®^ II Reverse Transcriptase system (Life Technologies Corp., Carlsbad, CA, USA). Quantification of mRNA was carried out using qPCR with SYBR green and the following primers: *Cox-2* (5′-CTGAGGGGTTACCACTTCCA-3′; and 5′-CTTGAACACGGACTTGCTCA-3′); *Il-1β* (5′-AGGCTTCCTTGTGCAAGTGT-3′ and 5′-TGAGTGACACTGCCTTCCTG-3′); *Vegf-a* (5′- GCCCATGAAGTGGTGAAGTT-3′ and 5′-ACTCCAGGGCTTCATCATTG-3′); and *Gapdh* (5′-CTCATGACCACAGTCCATGC-3′ and 5′-TTCAGCTCTGGGATGACCTT-3′). The cycling protocol was as follows: denaturation (95 °C for 10 min), 45 cycles of amplification (95 °C for 15 s, 59 °C for 15 s, and 72 °C for 20 s), and a final extension at 72 °C. A melting curve analysis was also performed to ascertain the specificity of the amplified product. The expression for each gene was normalized to *Gapdh*. Expression was quantified as fold-change using the ∆∆Ct method.

### 2.7. Statistical Analysis

We used GraphPad Prism software (La Jolla, CA, USA) (version 5.0). Values are mean with standard deviation (mean ± SD). In all cases, we used unifactorial analysis of variance followed by Tukey’s post-hoc analysis.

## 3. Results

### 3.1. Amelioration of Corneal Wound Repair

**Figure 1:**
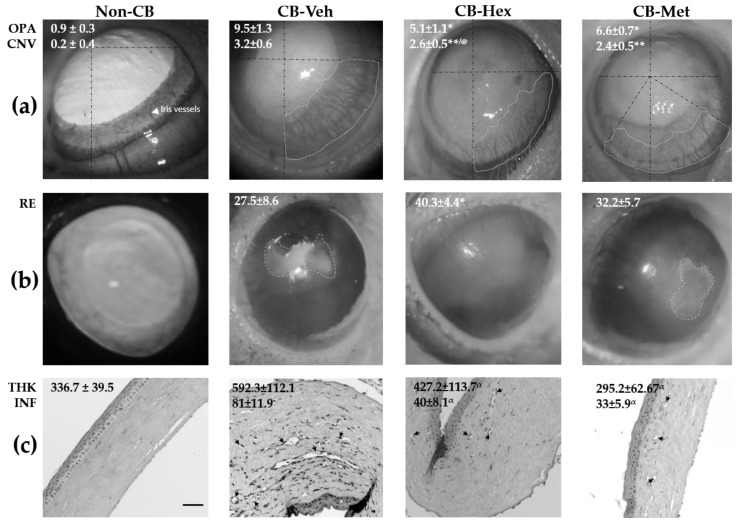
Methanol (Met) and hexane (Hex) extracts of *Cucurbita argyrosperma* seed in chemically burned corneas (CB) compared to the untreated group (CB corneas treated with the vehicle, CB-Veh). (**a**) Average of opacity score (OPA) and corneal neovascularization area (CNV) in mm^2^. (**b**) Re-epithelialization percentage (RE). (**c**) Corneal thickness in microns (THK) and infiltration cell percentage (INF). Average value ± SD; * *p* < 0.05; ** *p* < 0.01, and *^α^ p* < 0.001 compared to CB-Veh. ^@^ *p* < 0.05 compared to CB-Met. Gray dotted lines show studied area, and black lines are the geometrical axis. CB-Hex: CB corneas treated with hexanic extract; CB-Met: CB corneas treated with methanolic extract. Arrows indicate the lumen of stromal blood vessels. (Scale bar = 100 μm).

We evaluated corneal wound healing mediated by the extracts in the alkali-burn corneal model. Figure 1a shows that the treated groups had a significant reduction in corneal opacity score and CNV area compared to the CB-Veh (*p* < 0.05 and *p* < 0.001, respectively). However, CNV in the CB-Met treated group was lower than the CB-Hex group (*p* < 0.05) and furthermore did not show a significant increase in the percentage of re-epithelialization (Figure 1b).

Using HE-stained slides (Figure 1c), the non-CB group had an average corneal thickness of 336.7 ± 39.5 μm, with an intact epithelial and stromal layer. There were neither inflammatory cells nor blood vessels. Conversely, corneal integrity in the CB-Veh group was severely impaired, with a loss of the epithelial cell layers and disruption of stromal collagen fibers. The corneal thickness in this group was 592.3 ± 112.1 μm, with an average cell infiltration of 81 ± 11.9%. Corneal thickness in the CB-Hex group was 427.2 ± 113.7 μm, which was significantly reduced compared to the CB-Veh), with a reduction in cell infiltration (40 ± 8.1%). Similarly, the CB-Met group showed a decrease in cell infiltration (33.2 ± 5.9%) and corneal thickness (295.2 ± 62.67 μm) (*p* < 0.001) compared to CB-Veh. Corneal thickness in the CB-Met group was not significantly different from that of the non-CB group.

### 3.2. Anti-inflammatory Effect

**Figure 2:**
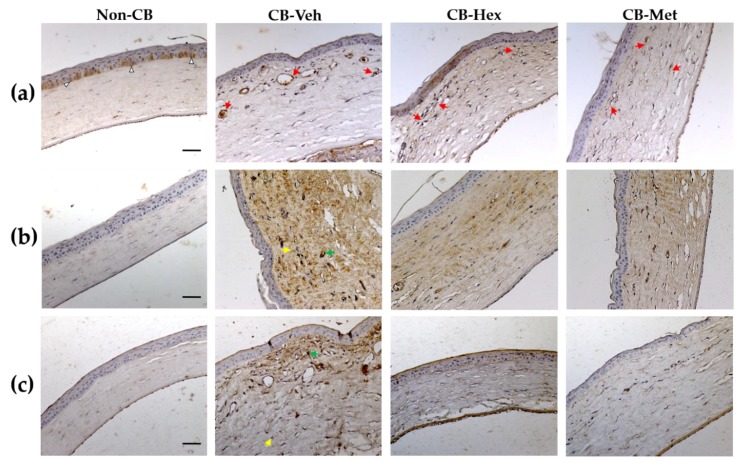
Micrograph of Met and Hex extracts of *C. argyrosperma* seed in the CB compared to the non-CB and CB-Veh groups. (**a**) Nuclei stained with anti-NF-κB p65 (red arrows). (**b**) Staining intensity for interleukin-1β (IL-1β) along with the corneal thickness. (**c**) Cyclooxigenase-2 (COX-2) staining in the studied groups. Arrowheads indicate the cytoplasmic distribution of NF-κB p65. Yellow and green arrows represent the minimum and maximum staining intensity, respectively, considered for software analysis. (Scale bar = 100 μm). CB: chemically burned corneas; CB-Veh: CB corneas treated with the vehicle; CB-Hex: CB corneas treated with hexanic extract; CB-Met: CB corneas treated with methanolic extract.

**Figure 3:**
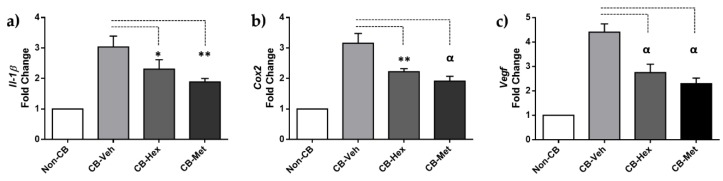
(**a**) qRT-PCR for *Il-1β*, (**b**) *Cox-2*, and (**c**) *Vegf-a*. Bars are the expression levels in each group. * *p* ≤ 0.05, ** *p* ≤ 0.01, and ^α^ *p* ≤ 0.001 compared to the CB-Veh group. CB: chemically burned corneas; CB-Veh: CB corneas treated with the vehicle; CB-Hex: CB corneas treated with hexanic extract; CB-Met: CB corneas treated with methanolic extract.

Several inflammatory cytokines implicated in alkali-induced corneal injury are regulated by the nuclear internalization of active NF-κB. Hence, we looked for the staining location of NF-κB in the corneal lesions. In the non-CB group, NF-κB was restricted to the cytoplasm of epithelial cells in the basal layer (Figure 2a). In the CB-Veh and both extract-treated groups, NF-κB was distributed in the nuclear compartment of endothelial and inflammatory cells. However, staining density decreased in the CB-Hex (41.13 ± 9.6) and the CB-Met (32.73 ± 8.1) compared to the CB-Veh (73.31 ± 10.4; *p* < 0.0001 both). Additionally, we performed measurements of the staining intensity for IL-1β (Figure 2b) and COX-2 (Figure 2c). In the non-CB, there was a low staining intensity for IL-1β (9.28 ± 2.6) compared to CB-Veh (75.95 ± 12.16; *p* < 0.0001), showing a distribution along with the corneal stroma as well as endothelial cells. Meanwhile, the intensities in the CB-Hex (42.16 ± 9.14) and the CB-Met (38.21 ± 7.9) were lower than the CB-Veh group (*p* < 0.0001). Staining intensity for IL-1β between the CB-Met and the CB-Hex showed no differences (*p* >0.05). Likewise, the intensity for COX-2 was significantly different when comparing the CB-Veh (102.6 ± 13.08) to the CB-Hex (68.79 ± 10.73) and the CB-Met (37.15 ± 7.18) (*p* < 0.0001) groups. The non-CB had a detectable expression of 7.49 ± 3.48. Staining intensity for IL-1β and COX-2 in the cornea was also confirmed at the level of mRNA (Figure 3): *Il-1β* expression for the CB-Met (2.31 ± 0.30) and the CB-Hex (1.89 ± 0.11) groups was decreased compared to the CB-Veh group (3.03 ± 0.35; *p* < 0.05 and *p* < 0.01, respectively) (Figure 3a). The *Cox-2* in the CB-Hex (2.22 ± 0.10) and the CB-Met (1.91 ± 0.15) was also diminished compared to the Veh group (3.15 ± 0.31; *p* < 0.01) (Figure 3b). The *Il-1ß* expression showed no difference between the CB-Met and the CB-Hex groups (*p* > 0.05); on the other hand, there were differences for *Cox-2* (*p* < 0.05).

### 3.3. Anti-Angiogenic Effect

**Figure 4:**
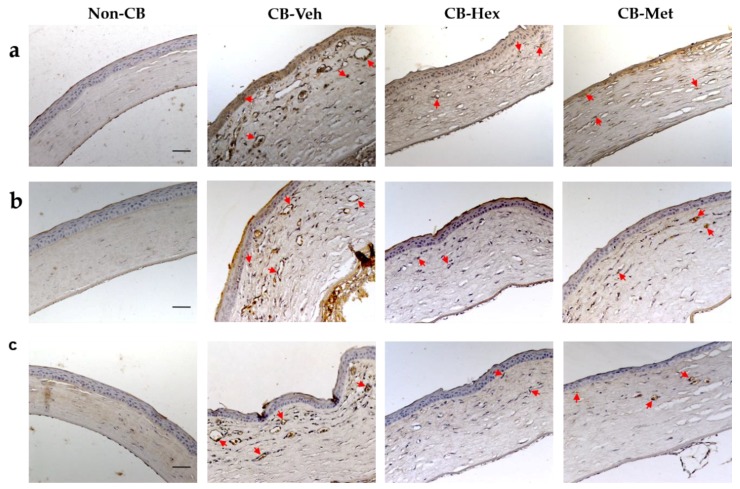
Immunolocalization of vascular endothelial growth factor A (VEGF-A) and its receptors, (vascular endothelial growth factor receptor 1, VEGFR1, and -receptor 2, VEGFR2), in the treated groups. (**a**) Staining intensities for VEGF-A. (**b**) and (**c**) Membranous staining in endothelial cells for VEGFR1 and VEGFR2, respectively (red arrows). Red arrows represent staining intensity considered for software analysis. (Scale bar = 100 μm). CB: chemically burned corneas; CB-Veh: CB corneas treated with the vehicle; CB-Hex: CB corneas treated with hexanic extract; CB-Met: CB corneas treated with methanolic extract.

Due to the anti-inflammatory effect in the CB-Hex and the CB-Met, we assessed whether it was related to an attenuation of CNV, by determining the staining intensity of VEGF-A and its receptors (Figure 4). In the CB-Veh, VEGF-A was in the cytoplasm and nucleus of the epithelial, endothelial, and other infiltrated cells with an intensity of 102.02 ± 14.04. A decrease in VEGF-A intensities was observed for the CB-Hex (71 ± 9.11) and CB-Met (61.3 ± 9.59) (*p* < 0.001) groups. Besides, staining intensity between VEGF-A in the CB-Met and CB-Hex groups showed differences (*p* < 0.05), and in the non-CB group it was about 16.25 ± 5.25 (Figure 4a). The *Vegf-a* expression was also confirmed (Figure 3c). The *Vegf-a* for the CB-Hex (2.74 ± 0.34) and CB-Met (2.29 ± 0.23) groups decreased compared to the CB-Veh (4.40 ± 0.34; *p* < 0.05 and *p* < 0.01, respectively).

Relevantly, staining for VEGFR1 was distributed in endothelial and inflammatory cells of the CB-Veh corneas (54.4 ± 6.8) and was higher when compared to the CB-Hex (33.13 ± 5.8; *p* < 0.0001) and CB-Met (25.47 ± 3.7; *p* < 0.0001) groups (Figure 4b). Vascular endothelial growth factor receptor 2 immunostaining was localized in the membrane region in endothelial cells, and in the CB-Veh (23.06 ± 3.5) it was higher compared to the CB-Hex (15.67 ± 2.6; *p* < 0.0001) and the CB-Met (10.95 ± 2.1; *p* < 0.0001) groups (Figure 4c).

## 4. Discussion

The cornea is a transparent, avascular, and immune-privileged tissue. However, the inflammatory response and growth of new vessels induced by infections, autoimmunity, and chemical burns may cause vision loss and a high rejection rate of corneal allografts if not treated effectively [1,25]. Recently, it has been demonstrated by histopathological and clinical observation in animal models that anti-neovascular topical treatments are successful at avoiding CNV [8,26]. To examine this phenomenon, experimental models combined with new therapeutic strategies have been used, primarily aimed at preserving corneal transparency through by attenuating the inflammatory and neovascularization responses.

Chemically burned corneas have long been used for this purpose because they are accompanied by the recruitment/migration of neutrophils and macrophages with resultant damage to the normal tissue structure. Furthermore, the release of oxidative derivatives, cytokines, chemokines, matrix metalloproteinases (MMPs), and growth factors including VEGF can influence corneal angiogenesis [21,27,28]. Topical treatments with monoclonal antibodies to VEGF-A or its receptor VEGFR2 suppress the mechanism of action of VEGF in endothelial cells, whereas dexamethasone inhibits CNV mediated by suppressing the activity of NF-κB, and decreasing the expression of IL-1β, COX-2, and VEGF [7,29]. In the same way, treatments with extracts of propolis and *Diospyros kaki*, as well as other purified phytochemicals (naringenin and (−)-Epigallocatechin gallate), have shown a decrease in CNV through down-regulation of VEGF-A, IL-1β, IL-6, and metalloproteases, promoting the healing of corneal wounds [12,13,14].

We aim to evidence the ability of the hexane and methanol extracts of *C. argyrosperma* seed to reduce CNV after an inflammatory stimulus induced by a chemical burn in rat cornea. To this end, we examined the nuclear localization of NF-κB p65, an important marker for the overexpression of VEGF-A, IL-1β, and COX-2 in damaged corneas. The receptor VEGFR2 in the endothelial cells of corneal stromal neovessels as an active marker of angiogenesis was studied. Angiogenesis is initiated when VEGFR2 is activated by tyrosine phosphorylation by VEGF-A binding. Consecutively, downstream pathways are activated, such as p38 MAPK and ERK1/2, producing a strong mitogenic and survival process [30,31]. In contrast, such a mitogenic signal is not equally induced by VEGFR1. Although VEGFR1 binds to VEGF-A with a higher affinity than VEGFR2, the induction of VEGFR1 phosphorylation is low and its downstream signaling is still poorly explored [32]. Vascular endothelial growth factor receptor 1 possesses anti-angiogenic activity by avoiding the union between VEGF-A and VEGR2. Corneal studies show that the proteolytic enzyme MMP14 can shave the extracellular domain of VEGFR1, converting it into a soluble receptor (sVEGFR) that acts as a decoy for VEGF-A [33]. Nevertheless, VEGFR1 indirectly induces angiogenesis by stimulating the migration of monocytes and macrophages directed towards the damaged microenvironment. For these reasons, this study evaluates both receptors because they are expressed predominantly in endothelial cells in the angiogenic environment: (1) VEGFR1 as a marker in the infiltration of monocytes and neovessels; and (2) VEGFR2 as an active marker of angiogenesis. Our results show that the staining intensity and expression of *Vegf-a* decreased along with the staining of VEGFR2 and VEGFR1 in endothelial cells of extract-treated corneas. Additionally, decreases in *Il-1β* and *Cox-2* expressions were also observed, suggesting that hexanic- and methanolic-extract components can attenuate these pro-angiogenic factors, likely through a lack of nuclear activation of NF-κB, which was also observed.

Treatment with hexanolic extract of *C. argyrosperma* seed shows a repairing of the corneal damage associated with a decrease in the expression of inflammatory and angiogenic factors. *Cucurbita pepo* seed extracts, containing mainly alpha-linoleic (ALA), linolenic acids (LA), tocopherols, and sterols, showed effective healing of skin wounds with a complete re-epithelialization, organization of collagen fibers, and absence of inflammatory cells [18]. Particularly, treatment with ALA in cultures of corneal epithelial cells and when it is topically applied to a dry eye animal model has anti-inflammatory activity, decreasing the release and expression of inflammatory factors (TNF-a, IL-6, IL-1β, and IL-8) regulated by the NF-κB pathway [34,35]. In addition, LA decreased corneal fluorescein staining and was associated with a significant decrease in the number of CD11b(+) cells [36].

Although we are still characterizing the bioactive compounds in the methanolic extract, we can speculate that the content of phytochemicals is similar to the *C. pepo* seed [16]. Some of these components include flavonoids such as quercetin, luteolin, and apigenin, which at low concentration intakes have protective anti-inflammatory effects on human retinal pigment epithelial damage by hypoxia, inhibiting VEGF and factors related to its activation [37]. For example, quercetin inhibits the production of inflammatory factors in VEGF-stimulated retinal photoreceptor cells, associated with inactivation of NF-κB as a consequence of the blockage of mitogen-activated protein kinases (MAPK) and protein kinase B (Akt) phosphorylation [38]. Nonetheless, *C. argyrosperma* may differ in the content and types of flavonoids from *C. pepo*. This would bring us closer to a better understanding of the mechanisms of attenuated angiogenesis by the phytochemicals contained in the methanol extract, which in turn could act synergistically, either directly or indirectly, in VEGF-A regulation.

Corneal inflammation eventually causes vision loss due to CNV. Corneal alkali-injury not only upregulated NF-κB, IL-1β, and COX-2 expression, it also significantly increased VEGF and its receptors VEGFR1 and VEGFR2 in endothelial cells. This work demonstrates for the first time, that methanolic or hexanic extracts of *C. argyrosperma* seed (400 mg/kg/7 days) improve the healing of corneal wounds caused by a chemical agent and may contribute to the anti-inflammatory properties of the phytochemicals in its composition. In addition, a significant reduction of the CNV was related to the attenuation of proangiogenic factors. Significantly, our results indicate that *C*. *argyrosperma* Hex extract is better than Met extract at reducing corneal re-epithelialization time, improving the healing process and thus preventing the entrance of microorganisms and inflammatory mediators into the deeper layers, probably through the inhibition of the NF-κB pathway for at least seven days after corneal alkali burn.

## 5. Conclusions

Intake of *Cucurbita argyrosperma* seed may be an option for preventing corneal angiogenesis. In addition, it might benefit wound healing or inhibit neovascularization in other degenerative pathologies. Further pharmacological and phytochemical studies are required to identify their constituents and accurately assess this activity.

## References

[1] Azar D.T. (2006). Corneal angiogenic privilege: Angiogenic and antiangiogenic factors in corneal avascularity, vasculogenesis, and wound healing (an American Ophthalmological Society thesis). Trans. Am. Ophthalmol. Soc..

[2] Kuwano T., Nakao S., Yamamoto H., Tsuneyoshi M., Yamamoto T., Kuwano M., Ono M. (2004). Cyclooxygenase 2 is a key enzyme for inflammatory cytokine-induced angiogenesis. FASEB J..

[3] Nakao S., Kuwano T., Tsutsumi-Miyahara C., Ueda S., Kimura Y.N., Hamano S., Sonoda K.H., Saijo Y., Nukiwa T., Strieter R.M. (2005). Infiltration of COX-2-expressing macrophages is a prerequisite for IL-1 beta-induced neovascularization and tumor growth. J. Clin. Investig..

[4] Sobolewski C., Cerella C., Dicato M., Ghibelli L., Diederich M. (2010). The role of cyclooxygenase-2 in cell proliferation and cell death in human malignancies. Int. J. Cell Biol..

[5] Cursiefen C., Chen L., Borges L.P., Jackson D., Cao J., Radziejewski C., D’Amore P.A., Dana M.R., Wiegand S.J., Streilein J.W. (2004). VEGF-A stimulates lymphangiogenesis and hemangiogenesis in inflammatory neovascularization via macrophage recruitment. J. Clin. Investig..

[6] Sivak J.M., Ostriker A.C., Woolfenden A., Demirs J., Cepeda R., Long D., Anderson K., Jaffee B. (2011). Pharmacologic uncoupling of angiogenesis and inflammation during initiation of pathological corneal neovascularization. J. Biol. Chem..

[7] Nakao S., Hata Y., Miura M., Noda K., Kimura Y.N., Kawahara S., Kita T., Hisatomi T., Nakazawa T., Jin Y. (2007). Dexamethasone inhibits interleukin-1beta-induced corneal neovascularization: Role of nuclear factor-kappaB-activated stromal cells in inflammatory angiogenesis. Am. J. Pathol..

[8] Gupta D., Illingworth C. (2011). Treatments for corneal neovascularization: A review. Cornea.

[9] Sheppard J.D., Comstock T.L., Cavet M.E. (2016). Impact of the Topical Ophthalmic Corticosteroid Loteprednol Etabonate on Intraocular Pressure. Adv. Ther..

[10] Al-Debasi T., Al-Bekairy A., Al-Katheri A., Al Harbi S., Mansour M. (2017). Topical versus subconjunctival anti-vascular endothelial growth factor therapy (Bevacizumab, Ranibizumab and Aflibercept) for treatment of corneal neovascularization. Saudi J. Ophthalmol..

[11] Zhou C., Robert M.C., Kapoulea V., Lei F., Stagner A.M., Jakobiec F.A., Dohlman C.H., Paschalis E.I. (2017). Sustained Subconjunctival Delivery of Infliximab Protects the Cornea and Retina Following Alkali Burn to the Eye. Investig. Ophthalmol. Vis. Sci..

[12] Keshavarz M., Mostafaie A., Mansouri K., Shakiba Y., Motlagh H.R. (2009). Inhibition of corneal neovascularization with propolis extract. Arch. Med. Res..

[13] Oguido A., Hohmann M.S.N., Pinho-Ribeiro F.A., Crespigio J., Domiciano T.P., Verri W.A., Casella A.M.B. (2017). Naringenin Eye Drops Inhibit Corneal Neovascularization by Anti-Inflammatory and Antioxidant Mechanisms. Investig. Ophthalmol. Vis. Sci..

[14] Yang S.J., Jo H., Kim K.A., Ahn H.R., Kang S.W., Jung S.H. (2016). Diospyros kaki Extract Inhibits Alkali Burn-Induced Corneal Neovascularization. J. Med. Food.

[15] Kocyan A., Zhang L.B., Schaefer H., Renner S.S. (2007). A multi-locus chloroplast phylogeny for the Cucurbitaceae and its implications for character evolution and classification. Mol. Phylogenet. Evol..

[16] Akomolafe S.F., Oboh G., Oyeleye S.I., Molehin O.R., Ogunsuyi O.B. (2016). Phenolic Composition and Inhibitory Ability of Methanolic Extract from Pumpkin (*Cucurbita pepo* L) Seeds on Fe-induced Thiobarbituric acid reactive species in Albino Rat’s Testicular Tissue In-Vitro. J. Appl. Pharm. Sci..

[17] Yadav M., Jain S., Tomar R., Prasad G.B., Yadav H. (2010). Medicinal and biological potential of pumpkin: An updated review. Nutr. Res. Rev..

[18] Bardaa S., Ben Halima N., Aloui F., Ben Mansour R., Jabeur H., Bouaziz M., Sahnoun Z. (2016). Oil from pumpkin (*Cucurbita pepo* L.) seeds: Evaluation of its functional properties on wound healing in rats. Lipids Health Dis..

[19] Medjakovic S., Hobiger S., Ardjomand-Woelkart K., Bucar F., Jungbauer A. (2016). Pumpkin seed extract: Cell growth inhibition of hyperplastic and cancer cells, independent of steroid hormone receptors. Fitoterapia.

[20] Sanchez-de la Vega G., Castellanos-Morales G., Gamez N., Hernandez-Rosales H.S., Vazquez-Lobo A., Aguirre-Planter E., Jaramillo-Correa J.P., Montes-Hernandez S., Lira-Saade R., Eguiarte L.E. (2018). Genetic Resources in the “Calabaza Pipiana” Squash (*Cucurbita argyrosperma*) in Mexico: Genetic Diversity, Genetic Differentiation and Distribution Models. Front. Plant. Sci..

[21] Choi H., Phillips C., Oh J.Y., Stock E.M., Kim D.K., Won J.K., Fulcher S. (2017). Comprehensive Modeling of Corneal Alkali Injury in the Rat Eye. Curr. Eye Res..

[22] Atiba A., Wasfy T., Abdo W., Ghoneim A., Kamal T., Shukry M. (2015). Aloe vera gel facilitates re-epithelialization of corneal alkali burn in normal and diabetic rats. Clin. Ophthalmol..

[23] Yoeruek E., Ziemssen F., Henke-Fahle S., Tatar O., Tura A., Grisanti S., Bartz-Schmidt K.U., Szurman P. (2008). Safety, penetration and efficacy of topically applied bevacizumab: Evaluation of eyedrops in corneal neovascularization after chemical burn. Acta Ophthalmol..

[24] Rogers M.S., Birsner A.E., D’Amato R.J. (2007). The mouse cornea micropocket angiogenesis assay. Nat. Protoc.

[25] Dana M.R., Streilein J.W. (1996). Loss and restoration of immune privilege in eyes with corneal neovascularization. Investig. Ophthalmol. Vis. Sci..

[26] Roshandel D., Eslani M., Baradaran-Rafii A., Cheung A.Y., Kurji K., Jabbehdari S., Maiz A., Jalali S., Djalilian A.R., Holland E.J. (2018). Current and emerging therapies for corneal neovascularization. Ocul. Surf..

[27] Shakiba Y., Mansouri K., Arshadi D., Rezaei N. (2009). Corneal neovascularization: Molecular events and therapeutic options. Recent Pat. Inflamm. Allergy Drug Discov..

[28] Edelman J.L., Castro M.R., Wen Y. (1999). Correlation of VEGF expression by leukocytes with the growth and regression of blood vessels in the rat cornea. Investig. Ophthalmol. Vis. Sci..

[29] Nakao S., Zandi S., Lara-Castillo N., Taher M., Ishibashi T., Hafezi-Moghadam A. (2012). Larger therapeutic window for steroid versus VEGF-A inhibitor in inflammatory angiogenesis: Surprisingly similar impact on leukocyte infiltration. Investig. Ophthalmol. Vis. Sci..

[30] Kim Y.M., Hwang S., Kim Y.M., Pyun B.J., Kim T.Y., Lee S.T., Gho Y.S., Kwon Y.G. (2002). Endostatin blocks vascular endothelial growth factor-mediated signaling via direct interaction with KDR/Flk-1. J. Biol. Chem..

[31] Gee E., Milkiewicz M., Haas T.L. (2010). p38 MAPK activity is stimulated by vascular endothelial growth factor receptor 2 activation and is essential for shear stress-induced angiogenesis. J. Cell Physiol..

[32] Koch S., Claesson-Welsh L. (2012). Signal transduction by vascular endothelial growth factor receptors. Cold Spring Harb. Perspect. Med..

[33] Han K.Y., Chang J.H., Lee H., Azar D.T. (2016). Proangiogenic Interactions of Vascular Endothelial MMP14 With VEGF Receptor 1 in VEGFA-Mediated Corneal Angiogenesis. Invest. Ophthalmol. Vis. Sci..

[34] Erdinest N., Shmueli O., Grossman Y., Ovadia H., Solomon A. (2012). Anti-inflammatory effects of alpha linolenic acid on human corneal epithelial cells. Investig. Ophthalmol. Vis. Sci..

[35] Erdinest N., Shohat N., Moallem E., Yahalom C., Mechoulam H., Anteby I., Ovadia H., Solomon A. (2015). Nitric oxide secretion in human conjunctival fibroblasts is inhibited by alpha linolenic acid. J. Inflamm..

[36] Rashid S., Jin Y., Ecoiffier T., Barabino S., Schaumberg D.A., Dana M.R. (2008). Topical omega-3 and omega-6 fatty acids for treatment of dry eye. Arch. Ophthalmol..

[37] Chen R., Hollborn M., Grosche A., Reichenbach A., Wiedemann P., Bringmann A., Kohen L. (2014). Effects of the vegetable polyphenols epigallocatechin-3-gallate, luteolin, apigenin, myricetin, quercetin, and cyanidin in primary cultures of human retinal pigment epithelial cells. Mol. Vis..

[38] Lee M., Yun S., Lee H., Yang J. (2017). Quercetin Mitigates Inflammatory Responses Induced by Vascular Endothelial Growth Factor in Mouse Retinal Photoreceptor Cells through Suppression of Nuclear Factor Kappa B. Int. J. Mol. Sci..

